# Supplementary material for: Rapid, Sensitive On-Site Detection of Deoxynivalenol in Cereals Using Portable and Reusable Evanescent Wave Optofluidic Immunosensor
Source: Int J Environ Res Public Health. 2022 Mar 22;19(7):3759. doi: 10.3390/ijerph19073759 (PMC8997826; doi:10.3390/ijerph19073759)
Supplement: Supplementary file 1 [file ijerph-19-03759-s001.zip › ijerph-1591161-supplementary.pdf]

International Journal of
Environmental Research and Public Health

Supporting Information:

Rapid, Sensitive On-Site Detection of Deoxynivalenol in Cereals Using Portable and Reusable Evanescent Wave Optofluidic Immunosensor

The ELISA method for DON detection

To achieve DON detection using ELISA, the coating antigen DON-BSA was diluted with sodium carbonate/bicarbonate buffer (0.1 mol·L^{-1}) to a concentration of 1.0 μg·mL^{-1}, and 100 μL of the solution was added to each well of a 96-well microtiter plate. Then, the plate was covered and incubated overnight at 4 °C (> 12 h). The wells were emptied, washed three times with PBST and dried. Each well was blocked with a 200 μL BSA solution (2%, w/v) for 2 h. The wells were subsequently emptied and washed again three times with a PBST. A 50 μL/well of DON solution of various concentrations, and a 50 μL/well of anti-DON antibody at the dilution ratio of 1:10000 was added. Inhibition standard curves were prepared with a DON concentration ranging from 0.01 to 1000 μg·L^{-1}. The competitive reaction was allowed to take place for 60 min. After washing, the peroxidase-labeled goat anti-mouse immunoglobulins at the dilution ratio of 1:1000 was added, and incubated for 30 min. Next, a 50 μL/well of freshly prepared tetramethylbenzidine (TMB) was added, and incubated for 15 min. The chemiluminescent emission of each well was measured after adding sulfuric acid for 10 s. All of the incubations were performed at 37 °C.

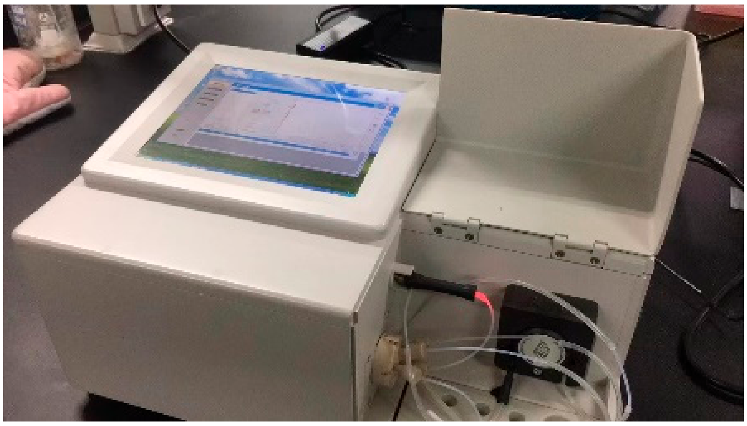

Figure S1. Photo of OIP-v2. The OIP-v2 has a size of 36×25×18cm and a weight of 2.5 kg.

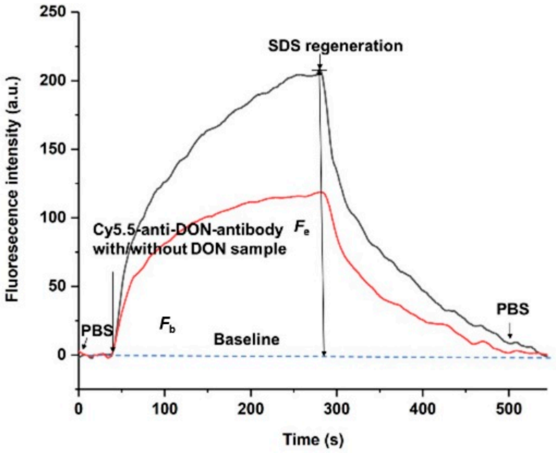

Figure S2. Feasibility of the DON-BSA functionalized bio-probe for DON detection. The concentration of the Cy5.5-anti-DON antibody and DON are 0.5 μg/mL and 100 μg/L, respectively.

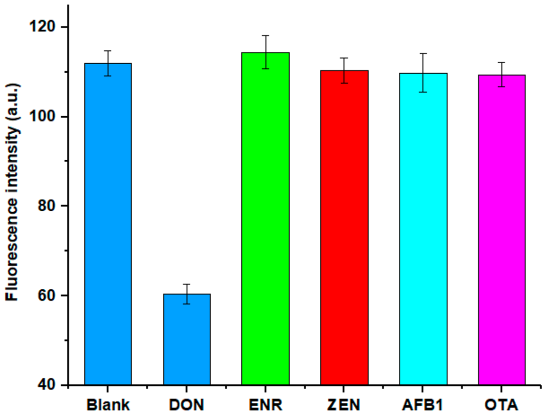

Figure S3. Selectivity of the Cy5.5-anti-DON antibody. The concentration of the anti-DON antibody is 0.25 μg·mL^{-1} and the concentration of DON, ENR, ZEN, AFB_1 and OTA is 10.0 μg·L^{-1}.

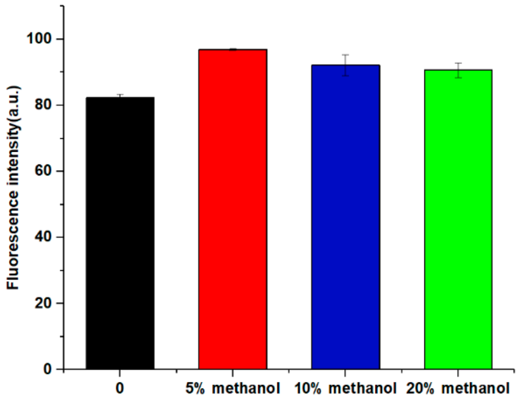

Figure S4. The effective fluorescence signal values at various methanol concentrations (0, 5%, 10%, 20% and v/v) without adding DON.

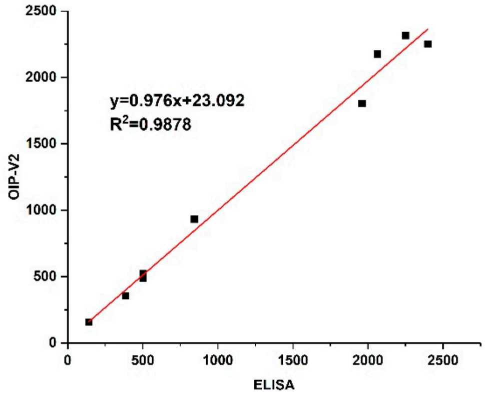

Figure S5. Comparison between the OIP-v2 and ELISA for the simultaneous detection of nine maize-positive samples.

Table S1. Comparison of analysis methods for DON and the OIP-V2 based assays.

Analytical Methods	Dynamic Detection Range (μg·L^{-1})	LOD (μg·L^{-1})	Reference
UPLC-MS	2-800	0.5	[1]
TRFIA	0.5-50	0.2	[2]
ELISA	1-100	0.56	[3]
FGN based ICS	5-20	5	[4]
Nanobody-based immunoassay	2.18–62.25	1.16	
Inhibition-based immunosensor	6-30	0.3	[5]
OIP-V2	0.43-36.61	0.16	This work

References

1. Zhang, J.; Sun, Y.; Zhao, L.; Gao, F.; Li, Y.; Ren, S. Comparative study of cation and anion ionization modes for detection of deoxynivalenol by ultra performance liquid chromatography-tandem mass spectrometry. *J. Food Saf. Qual.* **2017**, *8*, 3969–3973.
2. Jue, Z. Study on Rapidly Simultaneous Detection of Deoxynivalenol and Zearalenone. *China Biotechnol.* **2009**, *29*, 82–88.
3. Zhang, Y.Z. Establishment of the Direct Competition ELISA Method for Deoxynivalenol(DON). *J. Food Sci. Biotechnol.* **2012**, *31*, 28–32.
4. Huang, X.Y.; Huang, T.; Li, X.J.; ; Huang, Z.B. Flower-like gold nanoparticles-based immunochromatographic test strip for rapid simultaneous detection of fumonisin B_1 and deoxynivalenol in Chinese traditional medicine. *J. Pharm. Biomed. Anal.* **2019**, *177*, 112895.
5. Sunday, C.E.; Masikini, M.; Wilson, L.; Rassie, C.; Waryo, T.; Baker, P.G.L.; Iwuoha, E.I. Application on Gold Nanoparticles-Dotted 4-Nitrophenylazo Graphene in a Label-Free Impedimetric Deoxynivalenol Immunosensor. *Sensors* **2015**, *15*, 3854–3871.
